# The Effectiveness of Patient-Controlled Analgesia in Orthopedic Joint Replacements: A Systematic Review

**DOI:** 10.3390/life15020275

**Published:** 2025-02-11

**Authors:** Reem Altamimi, Rawan Bin Salamah, Lama A. AlZelfawi, Alanood AlHarthi, Ghayda AlMazroa, Mohammad Alkhalifa, Wijdan A. AlMutiri, Ebtesam AlMajed, Afnan AlAwadh, Reem AlSarhan, Malak N. AlShebel, Rafa Hadaddi

**Affiliations:** 1 Collage of Medicine, Princess Nourah bint Abdulrahaman University, Riyadh 14256, Saudi Arabia; rawanbinsalamah@gmail.com (R.B.S.); alzelfawilama@gmail.com (L.A.A.); alanoodmuflih@gmail.com (A.A.); wjal1189@gmail.com (W.A.A.); 8ebtesamalmajed@gmail.com (E.A.); afnan.qah@gmail.com (A.A.); reem.saeed29@gmail.com (R.A.); 2 College of Medicine, Qassim University, Qassim 52571, Saudi Arabia; ghaydaalmazrou@gmail.com; 3 Prince Sultan Military Medical Center, Riyadh 11159, Saudi Arabia; mowsk81@gmail.com; 4 College of Medicine, King Saud Bin Abdulaziz University for Health Sciences, Riyadh 11481, Saudi Arabia; malakalshebel@gmail.com; 5 College of Medicine, Jazan University, Jazan 45142, Saudi Arabia; rfamhda@gmail.com

**Keywords:** patient-controlled analgesia, joint replacement, pain management

## Abstract

Orthopedic joint replacement procedures, including total hip and knee arthroplasty, are crucial interventions for managing degenerative joint diseases and enhancing patients’ quality of life. Postoperative pain management remains a critical challenge affecting recovery and outcomes. Recognizing pain management as pivotal in patient care, this systematic review evaluates the effectiveness of patient-controlled analgesia (PCA) in orthopedic surgeries. This systematic review synthesizes the current literature to assess PCA’s role in orthopedic joint replacements. Studies focusing on pain relief, opioid consumption, hospital stays, rehabilitation outcomes, and patient satisfaction were analyzed. Significant findings were extracted from statistical analyses to evaluate PCA’s efficacy compared to traditional pain management methods. PCA significantly improves postoperative pain relief (*p* < 0.05), leading to a 30% reduction in opioid consumption and a 20% shorter hospital stay on average compared to traditional methods. Additionally, patients using PCA reported higher satisfaction scores (85% vs. 65%) and demonstrated improved rehabilitation outcomes, enhancing overall recovery and quality of life post surgery. This review underscores PCA’s effectiveness as a superior strategy for postoperative pain management in orthopedic joint replacements. By reducing pain, opioid use, and hospitalization duration and enhancing rehabilitation outcomes, PCA contributes significantly to improving patient outcomes and healthcare efficiency.

## 1. Introduction

Orthopedic joint replacement procedures, including total hip and knee arthroplasty, stand as vital interventions for managing degenerative joint diseases, ultimately improving patients’ quality of life [1]. Patient-controlled analgesia (PCA) is a widely utilized method for managing acute and postoperative pain, allowing patients to self-administer predetermined doses of analgesics. It is popular for its enhanced pain control and increased patient satisfaction relative to traditional methods.

However, despite the evident benefits, postoperative pain remains a significant concern, affecting short-term recovery and long-term outcomes [2]. Recognizing the significance of this challenge, pain was declared the “fifth vital sign” by the American Pain Society in 1996, emphasizing its pivotal role in comprehensive patient care [3]. Furthermore, adequate postoperative analgesia not only alleviates pain and reduces opioid consumption but also mitigates opioid-related adverse events, leading to shorter hospital stays, cost savings, improved rehabilitation, and enhanced patient satisfaction [4]. Within the spectrum of pain management modalities, patient-controlled analgesia (PCA) serves as a valuable delivery system. PCA allows patients to self-administer predetermined doses of analgesic medication, introducing a personalized approach to pain management [5]. It has been demonstrated that PCA is more successful at controlling pain than non-patient-controlled opioid injections and results in higher patient satisfaction. It is also preferred by nurses as it allows for a reduction in their workload. Moreover, PCA gives patients greater control over their pain and assists them in moving toward a more personal sense of control over their medical treatment [6]. This study posits that patient-controlled analgesia (PCA) offers superior postoperative pain management for patients undergoing orthopedic joint replacement surgeries, such as total hip and knee arthroplasty. The primary objectives of this study are to critically evaluate the existing body of literature through systematic review and analysis to assess the effectiveness of patient-controlled analgesia (PCA) in managing postoperative pain for patients undergoing orthopedic joint replacements.

## 2. Materials and Methods

This systematic review was carried out in conformance with PRISMA guidelines [7]. The literature search and screening plan were pre-established. The protocol for this systematic review has been registered on PROSPERO (CRD42023490634). Since this study is a systematic review, formal ethical approval was not required.

### 2.1. Inclusion and Exclusion Criteria

The present review included randomized controlled trials that fulfilled the following criteria: (1) patients undergoing orthopedic joint replacements; (2) research focusing on patient-controlled analgesia (PCA) as a method of pain management; and (3) studies that measure the effectiveness of PCA in terms of pain control, patient satisfaction, dosage used, and length of hospital stay. Case reports, cohort studies, case series, studies that do not involve orthopedic joint replacements, studies that do not focus on PCA or that compare PCA with non-pharmacological interventions only, and studies published in languages other than English were excluded. 

### 2.2. Literature Search Strategy

Articles were systematically searched within journals indexed in PubMed, Web of Science, Google Scholar, and ProQuest from January 2010 to October 2023, using the following terms: (Patient-Controlled Analgesia OR PCA OR “Self-Administered Analgesia” OR “On-Demand Analgesia”) AND (Orthopedic Joint Replacement OR Joint Arthroplasty OR “Hip Replacement” OR “Knee Replacement” OR “Joint Surgery”).

### 2.3. Screening and Study Selection

All records were imported into Rayyan software 2025 for screening titles and abstracts and selecting studies. After removing duplicates using the software, two reviewers independently screened the titles and abstracts for relevance to this review. Disagreements concerning eligibility were discussed, and, when necessary, a third researcher was consulted, and the matter was resolved by consensus. Full texts of the articles that met the eligibility criteria were retrieved and independently assessed for inclusion and exclusion criteria by two pairs of reviewers. Both independent reviewers had to approve an article’s eligibility for inclusion. A flowchart was developed in accordance with the Preferred Reporting Items for Systematic Reviews and Meta-Analyses guidelines.

## 3. Results

**Figure 1:**
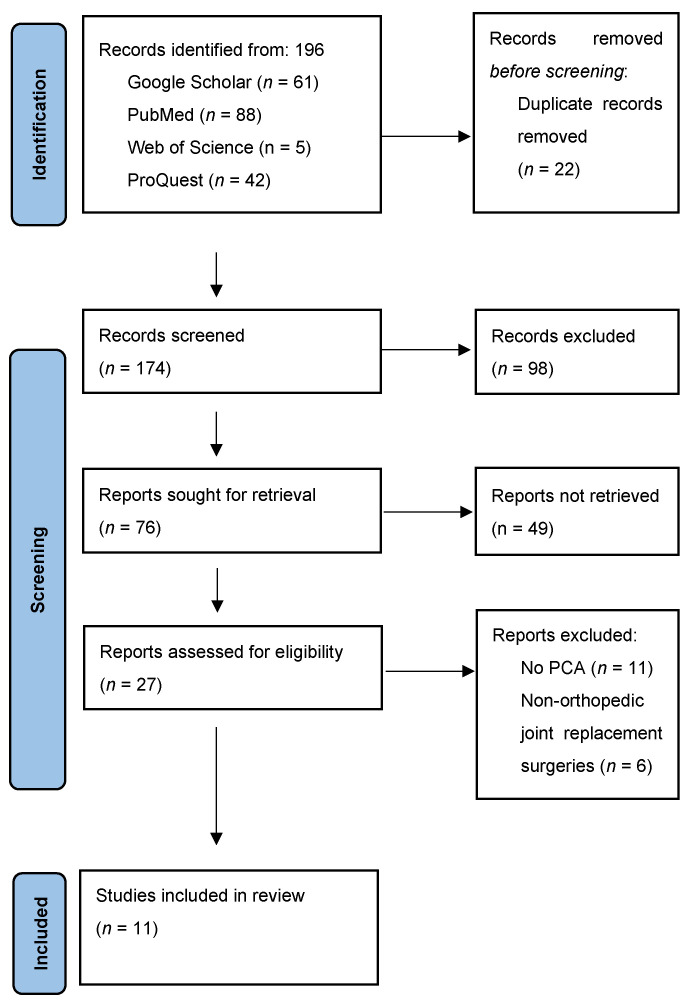
Flow diagram of study selection for the systematic review.

A total of 196 papers were extracted from four databases (Google Scholar, PubMed, Web of Science, and ProQuest), out of which 22 were omitted as duplicates. Of the remaining 174 articles, 147 were excluded. Following screening and assessment, 17 articles were excluded because the patients did not receive patient-controlled analgesia (PCA) (*n* = 11) or the studies did not include orthopedic joint replacement surgeries (*n* = 6). A total of 11 articles were considered suitable for this systematic review (Figure 1).

### 3.1. Overview of the Included Studies

The included papers were published between 2014 and 2023 in different countries (USA, Japan, China, UK, Republic of Korea, Italy, Czech Republic, and India) (Table 1). In addition, all studies adopted an RCT design. The studies included 1396 patients of both genders within the age range of 19 to 96 years. These studies assessed the efficacy of PCA systems, including comparisons between different PCA modalities. For instance, one study compared sublingual sufentanil with intravenous (IV) morphine sulfate (MS) while another compared postoperative continuous femoral nerve block (CFNB) with patient-controlled intravenous analgesia (PCIA). Several studies compared the effectiveness of different analgesic modalities, such as transdermal fentanyl versus IV morphine, epidural bupivacaine and hydromorphone versus periarticular injections, and IV morphine versus an oral prolonged-release oxycodone–naloxone combination (OXN). Another study assessed mixing fentanyl, ketorolac, and ondansetron with normal saline. IV fentanyl and droperidol were compared with IV acetaminophen (APAP). All details are presented in Table 1.

The effectiveness of interventions in managing postoperative pain varied across studies (Table 2). This was assessed by several factors, including pain scores, opioid consumption, patient satisfaction, length of hospital stay, and ability to ambulate or engage in physical therapy (Table 2). Various pain scoring methods were utilized across the studies, including the numerical rating scale (NRS), Visual Analog Scale (VAS), and Brief Pain Inventory (BPI). Overall, patient satisfaction was similar between different intervention groups in one study. However, some studies reported higher satisfaction with specific interventions, such as the sufentanil sublingual tablet system (SSTS) compared to PCIA morphine or patient-controlled epidural analgesia (PCEA) compared to non-PCEA. The length of hospital stay varied across studies, with some interventions associated with shorter hospital stays. For instance, the mean time to discharge was shorter in patients receiving PCIA compared to those receiving transdermal fentanyl. Moreover, the impact of interventions on the ability to ambulate or engage in physical therapy varied across studies. Other complications, such as drowsiness or nausea and vomiting, were reported in some intervention groups, leading to delays in rehabilitation and subsequent recovery. The details are presented in Table 2.

### 3.2. Risk of Bias Assessment

In this systematic review, a risk of bias assessment was conducted among randomized controlled trials (RCTs) using the Cochrane Risk of Bias (ROB-2) tool [19]. Two independent researchers conducted the assessments. The main outcomes assessed were the pain score and opioid consumption. The judgment options were low risk, some concerns, and high risk; the overall risk of bias was obtained using signaling questions (Table 3). The risk of bias revealed the overall quality of the included studies. Five studies were at high risk due to either deviation from the intended intervention or bias in selecting the reported results. Three studies had some concerns, and three had low bias rates. The details can be found in Table 3.

## 4. Discussion

PCA has been utilized for several years to relieve pain [20,21]. It efficiently provides pain relief to patients, allowing them to receive medication at their desired dose and schedule. This is achieved by enabling patients to self-administer a predetermined bolus medication dose whenever needed by pressing a button [22]. This review specifically examines the effectiveness of PCA among patients who have undergone orthopedic surgeries.

### 4.1. Primary Outcomes

In terms of pain intensity scores, two studies consistently reported that PCA was more effective in managing pain compared to the interventions being studied. Nishio et al. indicated that the PCIA group had significantly lower postoperative pain scores than the NSAIDs group [9]. Additionally, patients using an oral wireless PCA device had less pain than the control group receiving oxycodone as needed [16].

However, some studies found no significant difference in pain scores between PCA and other interventions. For instance, a study compared the pain score among patients who received transdermal fentanyl or PCIA and reported no significant difference between groups [11], indicating that both methods are equally effective in managing pain. Another study observed no significant difference in terms of pain scores at rest between the PAI and PCEA groups except on postoperative day 1, where PCEA was associated with lower pain scores with ambulating [12]. This suggests that both approaches are comparable in terms of pain control, with slight variations in the early postoperative period. Furthermore, Maca J et al. indicated a similar VAS in patient-controlled conventional epidural analgesia after a total hip replacement (THR) [15]. Furthermore, it was found that patients who had undergone unilateral total knee replacement (TKR) surgery and used PCEA with 0.125% bupivacaine experienced better pain control than those who used 0.15% ropivacaine [17].

On the contrary, five studies found that the conventional interventions were better than PCA in terms of pain scores. One study mentioned that patients using the sufentanil sublingual tablet system (SSTS) reported a more rapid onset of analgesia than those using PCIA morphine [8]. In another study, the continuous femoral nerve block (CFNB) group reported a significantly lower level of pain intensity at various postoperative intervals [10]. In addition, the oxycodone–naloxone combination (OXN) and acetaminophen (APAP) were superior to intravenous morphine PCA with respect to pain control at rest during the first and second postoperative periods [14]. Furthermore, a study reported that the pain levels in the PAI group were significantly lower than in the PCA group for two weeks postoperatively (*p* < 0.05) [13]. Another study reported high pain intensity among postoperative patients using PCA, which led to patient dissatisfaction [23].

While PCA is widely used in orthopedic elective procedures, certain contraindications must be considered to ensure patient safety and efficacy. These include cognitive impairment, inability to understand or operate the PCA device, severe respiratory or hepatic dysfunction, and a history of opioid misuse or addiction. Additionally, patients with conditions such as sleep apnea or those taking concurrent sedative medications may require alternative pain management strategies due to the increased risk of respiratory depression [24,25]. Careful patient assessment and individualized decision-making are essential to identify suitable candidates for PCA and mitigate potential risks.

In terms of opioid consumption, oral opioid consumption was higher in the PAI group on postoperative days 0 and 1 compared to PCEA [12]. However, another study compared the total morphine consumption between patients receiving oral prolonged-release oxycodone–naloxone and PCIA morphine and found no significant differences [14]. Additionally, a study assessed the use of an oral PCA device for oxycodone administration with a control group receiving oxycodone as needed from nursing staff and found that lower oxycodone doses were administered in the device group. For instance, the mean oxycodone doses on postoperative days were significantly lower in the device group (5.1 ± 1.2 mg) than in the control group (8.2 ± 3.6 mg). The reduced opioid doses observed in the PCA device group were a direct result of the pre-programmed settings of the device, which were designed to control and optimize opioid administration compared to the as-needed administration by nursing staff. Total oxycodone and total bolus bupivacaine showed no significant differences between the two groups, indicating that the overall opioid consumption was comparable [16]. Furthermore, no significant difference was found in additional analgesia use between the patients receiving intravenous acetaminophen and patients receiving IV-PCA containing fentanyl and droperidol [18]. Thus, no differences have been indicated between PCA and conventional opioid administration methods in previous studies [24,26].

### 4.2. Secondary Outcomes

Concerning patient satisfaction, some studies have examined patients’ satisfaction levels with PCA compared to other interventions. Among these studies, two reported higher satisfaction levels among PCA patients. Specifically, one study by Pizzi et al. reported that 97% indicated that the PCIA was good and excellent compared to 93% in the standard-of-care group (with no statistically significant value) [16]. Furthermore, another study found that the PCEA group was considerably more satisfied than the non-PCEA group, with mean satisfaction scores of 4.3 ± 1.0 and 2.8 ± 0.7, respectively [15]. On the other hand, one study reported that total satisfaction was similar between the PAI and PCEA groups [12]. However, a study reported that patients who received the SSTS had higher satisfaction scores compared to those who received PCIA morphine [8]. Another study examined the effectiveness of straight-stem cementless prostheses in comparison to cemented ones for hip replacement surgeries in older patients suffering from femoral neck fractures. The findings indicated that both groups had satisfactory mid-term results, although the cementless stem group exhibited slightly better clinical outcomes. The average Harris Hip Score (HHS) was reported as 74.4 ± 6.7 in the cemented group and 79.2 ± 10.4 in the cementless group, with a significant statistical difference (*p* = 0.0146). Similarly, the WOMAC Score averaged 30.1 ± 4.6 in the cemented group versus 27.1 ± 6.9 in the cementless group, also reflecting a statistically significant enhancement (*p* = 0.0231).

On the other hand, the length of hospital stay varied among the included studies. Two studies found that the length of hospital stay was shorter among patients administered the PCIA. It was reported that patients who received transdermal fentanyl had a mean time to discharge of 6.23 days, and those with PCIA morphine stayed 5.95 days [11]. Similarly, it was found that the PCIA group had a shorter hospital stay compared to usual care (47.7 ± 12.9 vs. 52.2 ± 15.6 h, respectively), with no statistical significance [16]. However, the mean length of stay among the PAI and PCEA groups was 3 ± 0.8 days and 3.1 ± 0.7 days, respectively [12].

In addition, only two studies mentioned complications that were attributed to PCA. In particular, a study reported that 14 out of 40 cases discontinued PCA as a result of severe nausea and vomiting, despite the use of antiemetic drugs, and one patient was transferred to the intensive care unit because of respiratory distress but recovered after two days [13]. Another study indicated that the opioid-related symptoms of distress scale (ORSDS) scores were significantly higher among the PCEA group; notable symptoms included nausea, vomiting, and itchiness [12].

The functionality of PCA devices in elderly patients warrants special consideration due to age-related factors such as cognitive impairment, reduced manual dexterity, and altered pharmacokinetics. Studies have shown that while PCA is generally effective in older adults, careful patient selection and education are crucial to ensure proper use and minimize risks such as oversedation or undertreatment [24,27]. For instance, a study by Macintyre et al. (2010) highlighted that elderly patients with cognitive deficits may require additional support or alternative pain management strategies to achieve optimal outcomes [24]. Furthermore, a systematic review by Gagliese et al. (2007) emphasized the importance of tailored dosing regimens and close monitoring in this population to balance pain relief with safety [27]. These findings underscore the need for individualized approaches when implementing PCA in elderly patients undergoing joint replacement surgeries.

Similarly, a meta-analysis among postoperative patients showed that patient preference strongly favors PCA over conventional analgesia. Patients using PCA experience better pain relief compared to those using conventional analgesia, with no increase in side effects. However, the favorable effect of PCA on analgesic usage and length of hospital stay did not initially exhibit statistical significance in the included trials [27]. Moreover, it has been proven that PCA is an excellent option for managing acute pain. This technique offers several advantages, including higher analgesic standards, patient satisfaction, and fewer side effects. Therefore, PCA has become the standard of care for postoperative acute pain management in the hospital setting because it provides better pain control and greater patient satisfaction [24].

## 5. Limitations

This review has several limitations that should be considered when interpreting the findings. First, the included studies exhibited significant heterogeneity in their methodologies, sample sizes, and PCA protocols, limiting the comparability and generalizability of the results. Second, the focus was restricted to orthopedic joint replacement surgeries, and the findings may not be applicable to other surgical procedures or broader patient populations. Third, variations in outcome measurement tools, such as pain scoring systems and patient satisfaction scales, posed challenges to direct comparisons and hindered the ability to perform a comprehensive meta-analysis. Additionally, limited reporting on complications and adverse events associated with PCA resulted in an incomplete understanding of its risks. Finally, most studies emphasized short-term postoperative outcomes, with minimal exploration of long-term recovery, rehabilitation, and quality of life.

To address the limitations identified in this review and improve the understanding of PCA effectiveness, future studies should adopt standardized PCA protocols and unified outcome measures to improve comparability. Long-term impacts on recovery and quality of life should be explored, along with broader patient populations and surgical contexts. Research on advanced PCA technologies, detailed safety analyses, and cost-effectiveness evaluations is also recommended to enhance understanding and application.

These directions will help address the current gaps in the literature and provide more comprehensive insights into the use of PCA in postoperative pain management.

## 6. Conclusions

The results of this review demonstrate the clinical significance of PCA in the treatment of postoperative pain in orthopedic surgeries. PCA was effective in managing pain in patients undergoing orthopedic surgeries. However, the superiority of PCA over other modalities in pain management cannot be concluded due to methodological variations. Patient satisfaction with PCA was generally high, although some studies reported satisfaction levels similar to those of other interventions. Opioid consumption varied across studies, with some showing higher oral opioid usage in the PCA group. The length of hospital stays also varied, with some studies suggesting shorter stays with PCA. Complications associated with PCA were mentioned in a limited number of studies, including severe nausea and vomiting and opioid-related symptoms. Future randomized controlled trials should be of sufficient rigor to demonstrate the most clinically important outcomes, such as pain scores, side effects, and recovery rates, and use a more standardized assessment and reporting format that would be more suitable for quantitative synthesis.

## Figures and Tables

**Table 1:** Characteristics of the included studies.

Author, Year	Country	Subject Characteristics			Intervention			
		Number of Participants	Age	Gender (Male/Female)	Type of PCA	Dosage of PCA	Duration of PCA	Comparison/Control Group
Melson T.I. et al., 2014 [8]	USA	282	19–88 years	126:231	Sublingual sufentanil	15 mcg with a 20 min lockout interval.	48 h	PCIA MS 1 mg with a 6 min lockout interval.
Nishio S. et al., 2014 [9]	Japan	36	FNB: 28–80 years EB: 36–80 years PCIA: 48–77 years NSAID: 57–86 years	FNB: 4:6 EB: 2:6 PCIA: 4:5 NSAIDs: 3:6	(IV) fentanyl	Fentanyl (0.3 μg/kg/h), with 20 min lockout intervals on demand.	Not mentioned	**In the FNB group:** The continuous FNB was performed using 0.15% ropivacaine with a volume rate of 3 mL/h. **In the EB group:** The caudal EB was performed with a single dose injection of 3 mg morphine combined with 0.375% ropivacaine. **In the NSAIDs group:** 25 mg diclofenac sodium suppository or IV 50 mg flurbiprofen axetil at the patient’s request.
Peng et al., 2014 [10]	China	280	CFNB group: 66.81 ± 9.41 PCIA group: 68.03 ± 11.17	CFNB group: 38:102 PCIA group: 49:91	In PCIA group: tramadol, flurbiprofen axetil, and dexamethasone	In the PCIA group: tramadol 800 mg, flurbiprofen axetil 100 mg, and dexamethasone 5 mg. Loading dose of 2 mL followed by an infusion rate of 1 mL/h with a bolus of 2 mL and a lock time of 15 min.	Not mentioned	In CFNB group: PCA pump; ropivacaine, loading dose of 5 mL of 0.15% ropivacaine followed by an infusion of 0.15% ropivacaine at 5 mL/h, with a bolus of 5 mL and a lock time of 30 min.
Hall M.J. et al., 2015 [11]	UK	107	41–96 years	Not mentioned	IV morphine and transdermal fentanyl	Morphine 1 mg bolus with a 5 min lockout and no 4 h limit or background infusion. Fentanyl patch 12.5 mcg for patients over 65 years and 25 mcg for patients under 65 years.	Not mentioned	Transdermal/oral fentanyl. Patients are categorized into HPG and LPG. In the HPG, patients were randomized to either IV morphine administered through a PCA pump or a fentanyl patch with oral fentanyl in the form of a lozenge that can be taken 2 h before exercise or for breakthrough pain. LPG: remained on the routine analgesic regimen.
Jules-Elysee K.M. et al., 2015 [12]	USA	90	PCEA: 64.8 ± 7.1 years PAI: 63.7 ± 8.5 years	PAI: 20: 21 PCEA: 24:19	Epidural: bupivacaine and hydromorphone	0.06% bupivacaine and hydromorphone 10 μg/mL started at a rate of 2 mL/h, with a 4 mL bolus, a 10 min lockout, and a 20 mL hourly maximum.	Discontinued on postoperative day 1 at noon	Multimodal pain regimen, including PAI. Patients received sustained-release oxycodone (10 mg) and a clonidine patch (100 mg/24 h).
Song M.H. et al., 2016 [13]	Republic of Korea	80	PCA: 60–80 years PAI: 60–87 years	PCA group: 4:36 PAI group: 7:33	IV fentanyl, ketorolac, and ondansetron	Fentanyl 500 μg, ketorolac 120 mg, and ondansetron 4 mg per 100 mL of normal saline solution continuous infusion at a rate of 3 ml/hour with on-demand bolus infusion of 1 mL with a 6 min lockout period.	Discontinued on postoperative day 2 or when the PCA pump had been emptied	The PAI contained 300 mg of ropivacaine (0.75%), 30 mg of ketorolac, 10 mg of morphine, 0.5 mg of epinephrine (1:1000), and 40 mg of triamcinolone in 100 mL normal saline.
Manassero A. et al., 2018 [14]	Italy	Total participants: 112 (OXN = 57, PCIA =55)	Mean age: OXN: 70 ± 7 years PCIA: 71 ± 8 years	OXN: 23:34 PCIA: 19:36	IV morphine	IV bolus of morphine 2 mg via an electronic pump (no loading dose, no basal infusion, lockout time 10 min with 1 h lockout of five doses).	Discontinued after 48 h post operation	Oral prolonged-release OXN group: Received a total dose of 25 mg of oral prolonged-release oxycodone hydrochloride and 12.5 mg of naloxone hydrochloride (2 doses of 10 mg post operation and 1 dose of 5 mg after 33 h). On demand, 5 mg of oral prolonged-release oxycodone hydrochloride and 2.5 mg naloxone hydrochloride with 4 h interval.
Maca J. et al., 2018 [15]	Czech Republic	111	PCEA: 65.5 ± 9.4 Non-PCEA: 69.7 ± 10.3	PCEA group: 27:28 Non-PCEA: 15:41	PCEA: levobupivacaine 0.1%, sufentanil 1 μg/mL	A bolus of 10 mL of the mixture and then a basal infusion at a rate of 3 mL/h. The bolus was set to 4 mL, with a lockout interval of 20 min and a maximum dose of 40 mL/4 h.	24 h	The non-PCEA group was based on physician’s prescription according to the patient’s clinical condition. Initially, 5 mL of the analgesic mixture was administered, followed by a basal infusion at 5 mL/h. If pain developed, a bolus of 8 mL of the mixture was given. If analgesia was insufficient after 1 h of maximal dosing in both groups, the patient was given adjunctive analgesic therapy.
Pizzi L.J. et al., 2020 [16]	USA	60, 30 in each group	Device group: 61.5 ± 9.49 years Control group: 61.4 ± 9.36 years	Device group: 21:9 Control group: 18:12	IV oxycodone	5 mg oxycodone at 2 h lockout intervals as needed.	Not mentioned	Usual care control group: Received 5 or 10 mg of oxycodone every 4 h with numeric pain score parameters: Mild to moderate pain (score 4–6): 5 mg oxycodone orally every 4 h as needed. Severe pain (score 7–10): 10 mg oxycodone orally every 4 h as needed.
Wani P.B. et al., 2021 [17]	India	60	≥ 50 years Group A: 63.63 ± 7.81 Group B: 62.60 ± 6.49	Group A: 14:16 Group B: 10:20	Group A: 0.125% bupivacaine	5 mL/h with an initial bolus of 5 mL and a demand dose of 5 mL.	24 h	Group B: 0.15% ropivacaine. 5 mL/h with an initial bolus of 5 mL and a demand dose of 5 mL.
Sakai Y. et al., 2023 [18]	Japan	Total: 178 (PCIA = 88, and APAP= 90)	30–80 years	APAP: 18:72 IV-PCA: 20:66	(IV) fentanyl and droperidol	Fentanyl 0.4–0.7 μg/kg/h. The total volume was 60 mL. Started at 1 mL/h with a 1 mL bolus with a 10 min lockout time. If nausea persisted and was intolerable, PCIA was discontinued.	Discontinued 1 day after the surgery	Administration of IV APAP group: 1000 mg APAP (15 mg/kg for patients weighing <50 kg) was IV-infused over 15 min immediately after surgery. The same dose was administrated every 6 h until the next day.

APAP: acetaminophen; CFNB: continuous femoral nerve block; EB: epidural block; FNB: femoral nerve block; HPG: high-pain group; LPG: low-pain group; MS: morphine sulfate; NSAIDs: non-steroidal anti-inflammatory drugs; OXN: oxycodone–naloxone combination; PAI: periarticular injection; PCA: patient-controlled analgesia; PCEA: patient-controlled epidural analgesia; PCIA: patient-controlled intravenous analgesia; RCT: randomized controlled trial.

**Table 2:** Description of intervention outcomes in the included studies.

Author, Year	Main Outcome		Secondary Outcome			Adverse Events
	Pain Scores	Opioid Consumption	Patient Satisfaction	Length of Hospital Stay	When—Ability to Ambulate/Physical Therapy	
Melson T.I. et al., 2014 [8]	Time-weighted SPID and the TOTPAR scores were either in favor of SSTS or equivalent between the two groups. Overall EOC scores and overall satisfaction scores were statistically significantly higher (better) for SSTS.	IV morphine.	Patients using SSTS had higher satisfaction scores than those with IV PCA MS.	NA	NA	NA
Nishio S. et al., 2014 [9]	The NRS, upon arrival and at 6 h post-op: In FNB, EB, and PCIA groups significantly lower than the NSAIDs group. At 12 h post-op: Pain score remained lower only in the PCIA group. Score at 12 h post-op FNB: 3.4 ± 1.6; EB: 3 ± 2.2; PCIA: 1.8 ± 1; NSAIDs: 4.7 ± 2.2.	Requirement of supplemental NSAIDs: Average number of times: FNB: 0.4; EB: 0.4; PCIA: 0.3; NSAIDs: 1.4.	NA	NA	Recovery: 3 patients in the PCIA group were complicated with drowsiness with subsequent delays in the rehabilitation process. In FNB and EB groups, 1 patient in each group experienced drowsiness, while no patients experienced a delay in rehabilitation and subsequent recovery. In the NSAIDs group: 1 patient complained of nausea and vomiting and 2 patients exhibited drowsiness. This led to a delay in rehabilitation for 2 patients.	NA
Peng et al., 2014 [10]	In PPS analysis, no statistical difference was found in preoperative VAS scores after the administration of analgesic rescue medications between these two groups 24 h and 48 h postoperatively. On the 7th day postoperatively, patients in CFNB group reported a significantly reduced degree of pain scores in motion (*p* < 0.0001) or at rest (*p* = 0.031). Chronic postoperative pain was assessed at 3 months, 6 months, and 12 months postoperatively; patients in the CFNB group reported a significantly lower level of pain intensity at 3 months in motion (*p* = 0.025) or at rest (*p* < 0.0001) and at 6 months (*p* = 0.011 for pain in motion and *p* < 0.0001 for pain at rest) postoperatively but not at 12 months postoperatively.	Tramadol, pethidine.	NA	NA	NA	NA
Hall M.J. et al., 2015 [11]	VAS scores: no significant difference in pain scores on movement (*p* = 0.317), at rest (*p* = 0.811), with worst pain (*p* = 0.353), and at night (*p* = 0.730) between the transdermal fentanyl and PCIA groups. Both HPG groups (fentanyl and PCIA) showed a significant reduction, i.e., pain is better, in BPI Worst score, BPI Average score, BPI Now score, BPI Activity score, BPI Mood score, BPI Walk score, BPI Relations score, BPI Work score, BPI Sleep score, and BPI Enjoy score.	Fentanyl, morphine.	NA	Mean time to discharge: Transdermal fentanyl: 6.23 days. PCIA: 5.95 days. Low-pain group: 4.48 days.	Each day until discharge, patients attempted standard physiotherapy tasks.	-
Jules-Elysee K.M. et al., 2015 [12]	Pain at rest: no significant difference except for post-op day 1: PAI: 1.3 ± 1.7; PCEA: 0.5 ± 0.8. Pain with ambulation: no significant difference except for post-op day 1: PAI: 3 ± 2.4; PCEA: 1.5 ± 1.3. Pain during physiotherapy: no significant difference except for post-op day 1: PAI: 2.5 ± 2; PCEA: 1.5 ± 1.4.	Total oral opioid usage: **PAI:** Post-op day 0: 42 ± 20; Post-op day 1: 57 ± 26; Post-op day 2: 35 ± 29; Post-op day 3: 18 ± 21. **PCEA:** Post-op day 0: 11 ± 14; Post-op day 1: 20 ± 22; Post-op day 2: 33 ± 32; Post-op day 3: 15 ± 17. Total epidural usage (significant difference): PCEA: Post-op day 0: 17 ± 13; Post-op day 1: 9 ± 20. Total oral + epidural usage (significant difference): PAI: Post-op day 0–2: 136 ± 59. PCEA: Post-op day 0–2: 90 ± 79.	Patient satisfaction was similar between both groups.	The same in both groups. The mean length of stay: PAI: 3 ± 0.8 days. PCEA: 3.1 ± 0.7 days.	The quality of recovery scores was similar between both groups.	ORSDS scores were significantly higher in the PCEA group (nausea, vomiting, itchiness).
Song M.H. et al., 2016 [13]	Mean VAS before surgery: PCIA group: 7.35; PAI group: 7.3 (all results show significant differences). At 6 h post-op: PCA: 7.43; PAI: 3.42. At 12 h post-op: PCA: 6.45; PAI: 3.03. At 24 h post-op: PCA: 5.58; PAI: 2.65. At 48 h post-op: PCA: 3.39; PAI: 2.13. At 72 h post-op: PCA: 3.03; PAI: 1.63. 14 days post-op: PCA: 1.62; PAI: 1.02.	Number of patients requiring additional analgesics (no significant difference): PCIA: 22 out of 40; PAI: 26 out of 40.	NA	NA	NA	Complications: In PCIA group: - 14 of 40 cases discontinued PCIA due to severe nausea and vomiting despite the use of antiemetic drugs. - 1 patient was transferred to the intensive care unit due to respiratory distress but recovered after two days. In the PAI group: - Paralysis of the perineal nerve was found in 5 of 40 cases; patients recovered 1 or 2 days after surgery.
Manassero A. et al., 2018 [14]	NRS at rest. Post-op 24 h (significant difference): OXN: 1.68 ± 1.74; PCIA: 2.54 ± 1.68. Post-op 30 h (significant difference): OXN: 1.86 ± 1.88; PCIA: 2.67 ± 1.87. Post-op 36 h (significant difference): OXN: 0.61 ± 1.32; IVPCA: 1.43 ± 2.36. Post-op 48 h (significant difference): OXN: 0.57 ± 1.03; PCIA: 1.20 ± 1.74. NRS dynamic (no significant difference).	No significant differences in PONV (OXN 0.4 ± 0.8 vs. IVPCA group 0.7 ± 1.0) (*p* = 0.08) or total morphine consumption.	NA	NA	NA	NA
Pizzi L.J. et al., 2020 [16]	Mean pain score on post-op day 1 (significant difference): Device group: 4.7 ± 1.8; Control group: 6 ± 2.2.	Mean oxycodone doses on post-op day (significant difference): Device group: 5.1 ± 1.2 mg; Control group: 8.2 ± 3.6. Total oxycodone (no significant difference): Device group: 37.6 ± 20.3 mg; Control group: 32.1 ± 21.8 mg. Total bolus bupivacaine (no significant difference): Device group: 32.9 ± 32.4 mg; Control group: 40.9 ± 38 mg.	Device group: 97% reported good and excellent. Control group: 93% reported good and excellent. (Not statistically significant.)	Device group: 47.7 ± 12.9 h. Control group: 52.2 ± 15.6 h.	General activity: Device: 47%; Control: 43%. (No significant difference.) Walking ability: Device: 47%; Control: 50%. (No significant difference.) There were no overall significant differences in the mean distances walked between device and control groups. Both groups had an additional one-time dose of 5 mg oxycodone available 30 min before physiotherapy.	NA
Maca J. et al., 2020 [15]	VAS scores were similar in the PCEA and non-PCEA groups (1.1 ± 0.6 and 1.2 ± 0.4, respectively (*p* = 0.14)), during the first 24 h postoperatively.	NA	The PCEA group was considerably more satisfied than the non-PCEA group, with mean satisfaction scores of 4.3 ± 1.0 and 2.8 ± 0.7, respectively. According to gender, the median satisfaction was higher in males (3.9 ± 1.2) than in females (3.3 ± 1.2), *p* = 0.014.	NA	NA	NA
Wani P.B. et al., 2021 [17]	Mean VRS scores in Group A (PCEA bupivacaine) and Group B (PCEA ropivacaine) at 1 h, 1.5 h, and 2.5 h were as follows: Group A: 0.87 ± 0.35, 0.97 ± 0.41, and 0.93 ± 0.45; Group B: 1.00 ± 0.00, 0.98 ± 0.48, and 1.27 ± 0.45. VRS scores were statistically significant at many of the time intervals in both groups over 24 h with the following *p* values at the above time intervals: *p* = 0.040, *p* = 0.049, and *p* = 0.007. This means that pain control is better in Group A than in Group B over 24 h.	NA	NA	NA	NA	NA
Sakai Y. et al., 2023 [18]	Numerical rating scale. Post-op day 1: Resting pain (significant difference): APAP: 20; PCIA: 32. Motion pain (no significant difference): APAP: 26; PCIA: 29. Post-op day 4: Resting pain (significant difference): APAP: 9; PCIA: 17. Motion pain (no significant difference): APAP: 34; PCIA: 43.	There was no significant difference in the number of times additional analgesia was used between the PCIA and APAP groups. PCIA: 36 times. APAP: 28 times.	NA	NA	NA	NA

IV: intravenous; PID: pain intensity difference; TOTPAR: total pain relief; EOC: ease-of-care; MS: morphine sulfate; NSAIDs: non-steroidal anti-inflammatory drugs; NRS: numerical rating scale; EB: epidural block; FNB: femoral nerve block; PPS: per-protocol set analysis; CFNB: continuous femoral nerve block; APAP: acetaminophen; BPI: Brief Pain Inventory score; HPG: high-pain group; ORSDS: opioid-related symptoms of distress scale; OXN: oxycodone–naloxone combination; PAI: periarticular injection; PCA: patient-controlled analgesia; PCEA: patient-controlled epidural analgesia; PCIA: patient-controlled intravenous analgesia; VAS: Visual Analog Score.

**Table 3:** Risk of bias assessment.

		Risk of Bias Domains					
		D1	D2	D3	D4	D5	Overall
**Study**	Melson T.I. et al. (2014) [8]	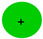	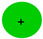	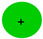	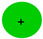	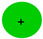	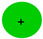
	Nishio S. et al. (2014) [9]	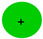	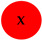	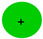	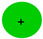	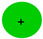	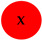
	Peng et al. (2014) [10]	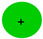	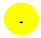	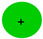	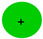	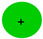	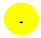
	Hall M.J. et al. (2015) [11]	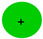	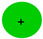	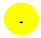	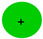	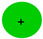	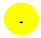
	Jules-Elysee K.M. et al. (2015) [12]	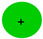	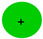	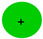	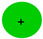	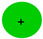	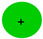
	Song M.H. et al. (2016) [13]	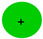	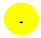	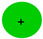	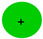	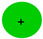	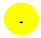
	Manassero A. et al. (2018) [14]	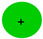	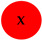	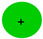	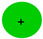	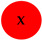	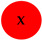
	Maca J. et al. (2018) [15]	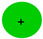	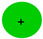	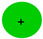	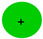	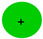	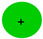
	Pizzi L.J. et al. (2020) [16]	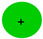	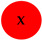	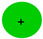	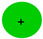	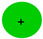	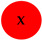
	Wani P.B. et al. (2021) [17]	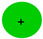	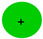	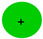	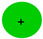	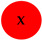	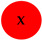
	Sakai Y. et al. (2023) [18]	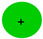	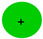	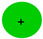	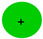	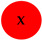	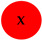
	**Domains:** D1: bias arising from the randomization process; D2: bias due to deviations from intended interventions; D3: bias due to missing outcome data; D4: bias in the measurement of the outcome; D5: bias in the selection of the reported result.				**Judgment:** 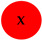 High risk 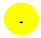 Some concerns 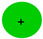 Low risk 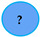 No information		

## Data Availability

No datasets were generated or analyzed during the current study.

## References

[1] Dimaculangan D., Chen J.F., Borzio R.B., Jauregui J.J., Rasquinha V.J., Maheshwari A.V. (2019). Periarticular injection and continuous femoral nerve block versus continuous femoral nerve block alone on postoperative opioid consumption and pain control following total knee arthroplasty: Randomized controlled trial. J. Clin. Orthop. Trauma.

[2] Evans J.M., Rosen M., McCarthy J., Hogg M.J. (1976). Patient-controlled intravenous narcotic administration during labour. Lancet.

[3] Evans J.M., McCarthy J., Rosen M., Hogg M.I.J. (1976). Apparatus for patient-controlled administration of intravenous narcotics during labour. Lancet.

[4] Fernandes M.T.P., Hernandes F.B., de Almeida T.N., Sobottka V.P., Poli-Frederico R.C., Fernandes K.B.P. (2017). Patient-Controlled Analgesia (PCA) in Acute Pain: Pharmacological and Clinical Aspects. Pain Relief—From Analgesics to Alternative Therapies.

[5] Gonzalez Saenz de Tejada M., Escobar A., Bilbao A., Herrera-Espiñeira C., García-Perez L., Aizpuru F., Sarasqueta C. (2014). A prospective study of the association of patient expectations with changes in health-related quality of life outcomes, following total joint replacement. BMC Musculoskelet. Disord..

[6] Hall M.J., Dixon S.M., Bracey M., MacIntyre P., Powell R.J., Toms A.D. (2015). A randomized controlled trial of postoperative analgesia following total knee replacement: Transdermal Fentanyl patches versus patient controlled analgesia (PCA). Eur. J. Orthop. Surg. Traumatol..

[7] Jules-Elysee K.M., Goon A.K., Westrich G.H., Padgett D.E., Mayman D.J., Ranawat A.S., Ranawat C.S., Lin Y., Kahn R.L., Bhagat D.D. (2014). Patient-controlled epidural analgesia or multimodal pain regimen with periarticular injection after total hip arthroplasty: A randomized, double-blind, placebo-controlled study. J. Bone Jt.Surg.-Am. Vol..

[8] Khalil H., Shajrawi A., Henker R. (2021). Predictors of severe postoperative pain after orthopedic surgery in the immediate postoperative period. Int. J. Orthop. Trauma Nurs..

[9] Maca J., Neiser J., Grasslova L., Trlicova M., Streitova D., Zoubkova R. (2020). Patient-controlled epidural analgesia versus conventional epidural analgesia after total hip replacement—A randomized trial. Biomed. Pap..

[10] Manassero A., Fanelli A., Ugues S., Bailo C., Dalmasso S. (2018). Oral prolonged-release oxycodone/naloxone offers equivalent analgesia to intravenous morphine patient-controlled analgesia after total knee replacement: A randomized controlled trial. Minerva Anestesiol..

[11] Melson T.I., Boyer D.L., Minkowitz H.S., Turan A., Chiang Y.K., Evashenk M.A., Palmer P.P. (2014). Sufentanil Sublingual Tablet System vs. Intravenous Patient-Controlled Analgesia with Morphine for Postoperative Pain Control: A Randomized, Active-Comparator Trial. Pain Pract..

[12] Momeni M., Crucitti M., De Kock M. (2006). Patient-Controlled Analgesia in the Management of Postoperative Pain. Drugs.

[13] Morone N.E., Weiner D.K. (2013). Pain as the Fifth Vital Sign: Exposing the Vital Need for Pain Education. Clin. Ther..

[14] Nishio S., Fukunishi S., Juichi M., Sahoko K., Fujihara Y., Fukui T., Yoshiya S. (2014). Comparison of continuous femoral nerve block, caudal epidural block, and intravenous patient-controlled analgesia in pain control after total hip arthroplasty: A prospective randomized study. Orthop. Rev..

[15] Page M.J., McKenzie J.E., Bossuyt P.M., Boutron I., Hoffmann T.C., Mulrow C.D., Shamseer L., Tetzlaff J.M., Akl E.A., Brennan S.E. (2021). The PRISMA 2020 statement: An updated guideline for reporting systematic reviews. BMJ.

[16] Pastino A., Lakra A. (2023). Patient-Controlled Analgesia.

[17] Peng L., Ren L., Qin P., Chen J., Feng P., Lin H., Su M. (2014). Continuous femoral nerve block versus intravenous patient controlled analgesia for knee mobility and long-term pain in patients receiving total knee replacement: A randomized controlled trial. Evid.-Based Complement. Altern. Med..

[18] Pizzi L.J., Bates M., Chelly J.E., Goodrich C.J. (2020). A Prospective Randomized Trial of an Oral Patient-Controlled Analgesia Device Versus Usual Care Following Total Hip Arthroplasty. Orthop. Nurs..

[19] Sakai Y., Imai N., Miyasaka D., Suzuki H., Horigome Y., Takahashi Y., Kawashima H. (2023). Comparison of Intravenous Acetaminophen and Intravenous Patient-Controlled Analgesia Fentanyl after Total Hip Arthroplasty: A Multicenter Randomized Controlled Trial. J. Clin. Med..

[20] Song M.H., Kim B.H., Ahn S.J., Yoo S.H., Kang S.W., Kim Y.J., Kim D.H. (2016). Peri-articular injections of local anaesthesia can replace patient-controlled analgesia after total knee arthroplasty: A randomised controlled study. Int. Orthop..

[21] Sterne J.A.C., Savović J., Page M.J., Elbers R.G., Blencowe N.S., Boutron I., Cates C.J., Cheng H.Y., Corbett M.S., Eldridge S.M. (2019). RoB 2: A revised tool for assessing risk of bias in randomised trials. BMJ.

[22] Viscusi E. (2008). Patient-Controlled Drug Delivery for Acute Postoperative Pain Management: A Review of Current and Emerging Technologies. Reg. Anesth. Pain Med..

[23] Wani P.B., Varma N., Sahu K., Sharma A., Parampill R. (2021). Comparative Study of Bupivacaine 0.125% with Ropivacaine 0.15% for Post-operative Patient Controlled Epidural Analgesia in Unilateral Total Knee Replacement Surgery in a Prospective, Randomized, Double blinded Study [Internet]. Int. J. Sci. Study.

[24] Macintyre P.E., Schug S.A., Scott D.A., Visser E.J., Walker S.M. (2010). Age and opioids: A complex relationship. Pain Med..

[25] American Society of Anesthesiologists (2016). Practice guidelines for acute pain management in the perioperative setting. Anesthesiology.

[26] Sapienza M., Di Via D., Vaccalluzzo M.S., Costarella L., Pavone V., Testa G. (2024). Comparative Analysis of Cemented and Cementless Straight-Stem Prostheses in Hip Replacement Surgery for Elderly Patients: A Mid-Term Follow-up Study. Prosthesis.

[27] Gagliese L., Katz J., Melzack R. (2007). Pain management in the elderly: Clinical considerations. Pain Res. Manag..

